# The P4-ATPase ATP9A is a novel determinant of exosome release

**DOI:** 10.1371/journal.pone.0213069

**Published:** 2019-04-04

**Authors:** Jyoti Naik, Chi M. Hau, Lysbeth ten Bloemendaal, Kam S. Mok, Najat Hajji, Ann M. Wehman, Sander Meisner, Vanesa Muncan, Nanne J. Paauw, H. E. de Vries, Rienk Nieuwland, Coen C. Paulusma, Piter J. Bosma

**Affiliations:** 1 Amsterdam University Medical Centers, university of Amsterdam, Tytgat Institute for Liver and Intestinal Research, Amsterdam Gastroenterology and Metabolism, Amsterdam, The Netherlands; 2 Laboratory of Experimental Clinical Chemistry, Vesicle Observation Centre, Amsterdam University Medical Centers, Academic Medical Center at the University of Amsterdam, Amsterdam, The Netherlands; 3 Rudolf Virchow Center for Experimental Biomedicine, University of Würzburg, Würzburg, Germany; 4 Department of Molecular Cell Biology and Immunology, Amsterdam University Medical Centers, VU University Medical Center, Amsterdam, The Netherlands; George Mason University, UNITED STATES

## Abstract

Extracellular vesicles (EVs) released by cells have a role in intercellular communication to regulate a wide range of biological processes. Two types of EVs can be recognized. Exosomes, which are released from multi-vesicular bodies upon fusion with the plasma membrane, and ectosomes, which directly bud from the plasma membrane. How cells regulate the quantity of EV release is largely unknown. One of the initiating events in vesicle biogenesis is the regulated transport of phospholipids from the exoplasmic to the cytosolic leaflet of biological membranes. This process is catalyzed by P4-ATPases. The role of these phospholipid transporters in intracellular vesicle transport has been established in lower eukaryotes and is slowly emerging in mammalian cells. In *Caenorhabditis elegans* (C. elegans), deficiency of the P4-ATPase member TAT-5 resulted in enhanced EV shedding, indicating a role in the regulation of EV release. In this study, we investigated whether the mammalian ortholog of TAT-5, ATP9A, has a similar function in mammalian cells. We show that knockdown of ATP9A expression in human hepatoma cells resulted in a significant increase in EV release that was independent of caspase-3 activation. Pharmacological blocking of exosome release in ATP9A knockdown cells did significantly reduce the total number of EVs. Our data support a role for ATP9A in the regulation of exosome release from human cells.

## Introduction

Extracellular vesicles (EVs) are carriers of a wide range of signaling molecules, including proteins, messenger- and micro-RNAs, that regulate a wide range of (patho)physiological processes, including blood coagulation, angiogenesis, detoxification and immune responses [1–4]. For instance, cancer cells use EVs to dictate their microenvironment to promote their proliferation and survival [5]. In addition, EVs are used by cells to selectively externalize proteins, such as the transferrin receptor during the maturation of reticulocytes [6]. Furthermore, drug transport by extracellular vesicles underlies multidrug resistance in cancer cells and to dispose of active caspase-3 thereby preventing apoptosis [7, 8]. Two classes of EVs (sizes ranging from 50–1000 nm) can be distinguished, i.e. exosomes and ectosomes, which differ in their route of secretion [9, 10]. Exosomes are released by fusion of multivesicular endosomes (MVEs) with the plasma membrane, whereas ectosomes are formed by direct outward budding of the plasma membrane [11].

Phospholipid asymmetry has long been implicated in vesicle release. Biological membranes consist of two leaflets of phospholipids that differ in composition. Phosphatidylserine (PS) and phosphatidylethanolamine (PE) species are almost exclusively present in the cytosolic leaflet, while phosphatidylcholine (PC) and sphingomyelin are enriched in the exoplasmic leaflet [12]. The asymmetric distribution of phospholipids is essential for cellular physiology and guarantees optimal membrane barrier function, membrane protein transport and signaling processes.

Several families of transporters actively maintain lipid asymmetry [13–15]. Members of the P4-ATPase family have been identified as lipid flippases [16, 17]. These proteins are involved in creating and maintaining lipid asymmetry in cellular membranes by moving lipids from the exofacial to the cytofacial leaflet. Accumulating evidence supports an important function for P4-ATPases in the biogenesis of transport vesicles in the endocytic and biosynthetic pathways in eukaryotic cells [15, 18]. A local concentration of lipids in one leaflet of the bilayer induces curvature of the membrane that serves as a binding scaffold for proteins of the trafficking machinery, which drives the initiation vesicle formation [19]. Recently, Wehman *et al*. demonstrated a role for the *C*. *elegans* P4-ATPase TAT-5 in the release of EVs and the maintenance of PE asymmetry [20]. TAT-5 deficiency resulted in excessive extracellular vesicle release and disrupted cell adhesion and gastrulation in *C*. *elegans* embryos. Their data provided the first evidence for a role of a P4-ATPase in the regulation of EV release [20]. While TAT-5 is expressed at the plasma membrane, the human orthologs of TAT-5, ATP9A and ATP9B are predominantly localized to intracellular compartments [21]. ATP9A has been reported to have a role in the endocytic recycling of plasma membrane proteins [22], but no one has tested whether ATP9A also regulates EV release. Given the (patho-)physiological importance of EVs in intercellular communication, clarification of the mechanisms that regulate EV release is of particular interest. Therefore, in the present study the role of ATP9A in the release of EVs from human cells was investigated.

## Results

### Depletion of ATP9A enhances EV release in HepG2 cells

**Fig 1:**
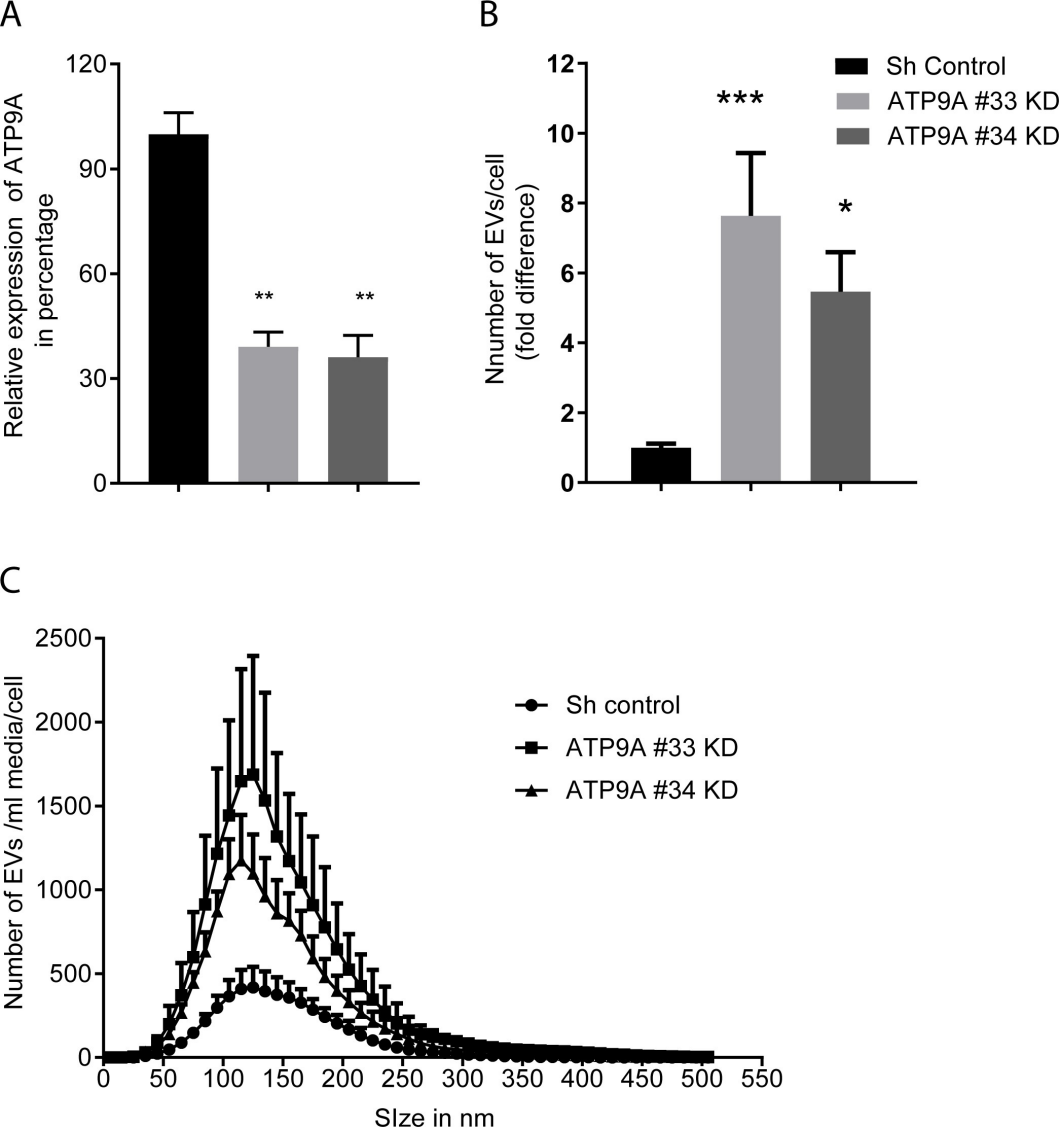
ATP9A knock-down in HepG2 cells enhances EV release. HepG2 cells were transduced with shATP9A #33, #34 or sh-control. Cells and medium were harvested 116 hours after transduction. (A) Ratio of *ATP9A* mRNA / geometric mean of *HRPT* and *36B4*. Data were presented as mean of 8 independent experiments ± standard error of mean (SEM). (B) Number of EVs/ml/cell in medium of *ATP9A* knock-down and sh-control cells. Values were expressed as mean of 8 independent experiments ± standard error of mean (SEM). (C) Size distribution of EVs in culture medium of sh control cells and ATP9A knock-down cells. For statistical significance One way ANOVA with Dunnett’s multiple comparison test was done. * P<0.05, **p<0.005,***P < 0.0005.

Loss of the P4-ATPase TAT-5 resulted in increased EV release from *C*. *elegans* embryos [20]. To investigate whether its mammalian ortholog ATP9A also controls EV release in mammalian cells, we studied the effect of *ATP9A* knock-down (KD) in the human hepatoma cell line HepG2. We chose to do our studies in HepG2 cells as ATP9A is abundantly expressed in mouse and human liver tissue (J. Naik, manuscript in preparation). Since constitutive depletion of *ATP9A* resulted in cell death after 10–14 days, the generation of a stable KD cell line was not feasible, hence ATP9AKD cells were generated before each experiment (detail in the material and methods). Because of lethality, we studied the consequence of ATP9A depletion up to 5 days post- transduction. Using lentiviral vectors expressing shRNA directed towards *ATP9A*, the mRNA expression of *ATP9A* was reduced by ~60% compared to cells transduced with a scrambled control shRNA (Fig 1A). Knock-down of *ATP9A* coincided with a 5 to 8-fold enhanced presence of EVs in the medium of two independent knock-down lines (Fig 1B). *ATP9A* depletion did not affect the size distribution of the EVs found in the medium (Fig 1C), which was consistent with increased EVs release. To provide evidence for a general role of ATP9A in the release of EVs, we quantified EV numbers in 2 other cell lines of different origin, i.e. the human breast cancer cell line MCF-7 and the human monocytic leukemia cell line THP-1. *ATP9A* knock-down in both THP-1 and MCF-7 cells also resulted in an increased number of EVs present in the medium (S1A–S1D Fig). These data indicate that ATP9A plays an important role in controlling the release of EVs from several human cell lines. Given the conserved function of TAT-5 and ATP9A in EV release, we tried to complement the phenotype of *ATP9A*-depleted HepG2 cells by over-expressing *tat-5* cDNA, however no protein expression was detected. Similarly, we attempted to complement the *C*. *elegans tat-5* mutant with ATP9A expression. We generated 121 *pie-1*-driven *GFP*:*ATP9A* cDNA strains, however we did not detect GFP expression in the germ line or early embryos. Regardless, our data suggests that TAT-5 and ATP9A have a conserved role inhibiting EVs release.

### EV release by ATP9A depleted cells is independent of caspase activation

**Fig 2:**
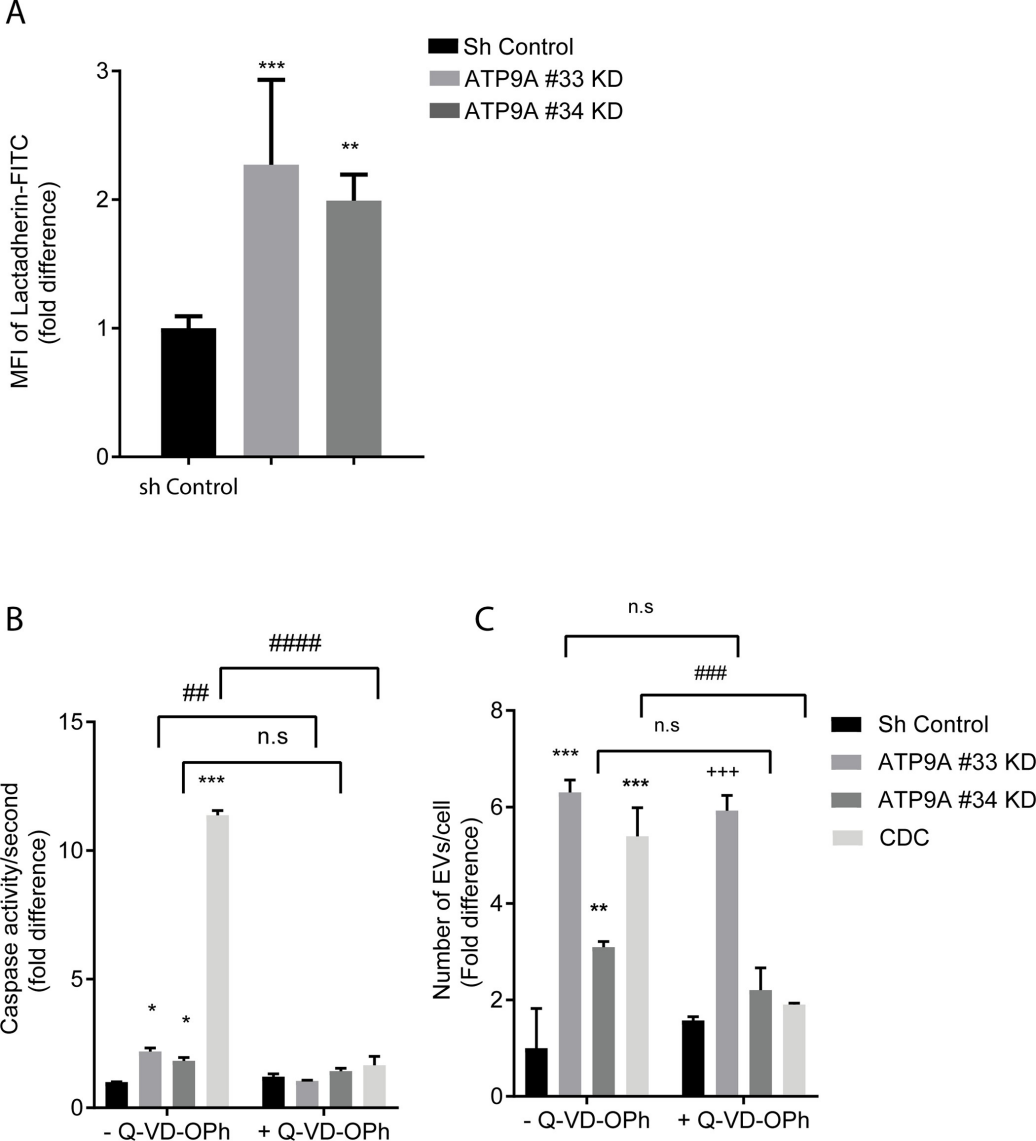
Enhanced EV release in ATP9A knock-down cells is independent of caspase-3 activation. (A) 116 hours after transduction, sh control and ATP9A knock-down cells were incubated with lactadherin-FITC and binding was analysed by FACS. Mean fluorescent intensity (MFI) of ATP9A knock-down cells compared to sh-control cells of two independent experiments performed in six replicates (n = 12) is presented. Statistical significance was tested by one-way ANOVA with Dunnett’s correction for multiple testing: **p<0.005,***p< 0.0005. (B) Caspase activity in ATP9A knock-down and Chenodeoxycholate (CDC) treated cells compared to sh-control cells in presence and absence of caspase inhibitor. Approximately 48 hours after transduction, sh-control and ATP9A knock-down cells were incubated with or without 5 μM caspase inhibitor (Q-VD-Oph) and 66 hours later caspase activity was determined. CDC was added as a positive control for apoptosis 1 hour before harvesting cells. (C) Number of EVs/cell in medium of ATP9A knock-down cells and CDC treated cells compared to sh-control cells. Values are expressed as the mean ± Standard deviation (SD) of 3 independent experiments. Statistical analysis was done by two-way ANOVA with Tukey’s correction for multiple testing: * represents the significant difference between the control and ATP9A knock-down or CDC treated cells in the vehicle treated groups;*p < 0.05, **, ***<0.0005. # shows the significant difference between the Q-VD-Oph treated and untreated groups; ##p<0.005, ###p<0.0005, n.s p≥0.05.+ represents the nonsignificant difference between control and ATP9A knock-down or CDC treated cells in the Q-VD-Oph treated groups; +++p <0.0005.

As *ATP9A*-depleted HepG2 cells stopped growing and started dying after prolonged culture, we investigated the possible contribution of apoptosis to the increased EV release 5 days post-transduction with *ATP9A* shRNAs. It has been known that apoptotic cell death is accompanied by EV release [23], although apoptotic vesicles are larger (>1000 nm) than the vesicles we observed upon *ATP9A* depletion (Fig 1C). We first tested whether HepG2 cells showed signs of apoptosis after 5 days of *ATP9A* depletion, such as phosphatidylserine exposure and caspase activation [24]. Significantly more lactadherin-FITC was observed in *ATP9A*-depleted cells compared to the controls, indicating increased phosphatidylserine in the exoplasmic leaflet of the cell membrane (Fig 2A). PS exposure was accompanied by a minor 1.3 to 2-fold increase in caspase-3/7 activity in *ATP9A* knock-down cells (Fig 2B), in comparison to the 11-fold increase in caspase activity observed with chenodeoxycholate (CDC) treatment, an established apoptotic trigger. We next investigated whether this minor increase in caspase activity was responsible for the enhanced EV release seen in *ATP9A* knock-down cells. Preincubation with the caspase inhibitor Q-VD-OPh prevented induction of caspase 3/7 activity in CDC-treated cells and in *ATP9A* knock-down cells (Fig 2B). Notably, inhibiting caspase activation normalized EV release from CDC-treated HepG2 cells, but failed to block the increased EV release from *ATP9A*-depleted cells (Fig 2C). This shows that the enhanced release of EVs by *ATP9A*-depleted cells is independent of caspase activation, and is suggesting a direct role of ATP9A in the release of EVs.

### ATP9A localizes in intracellular compartments and at the plasma membrane

**Fig 3:**
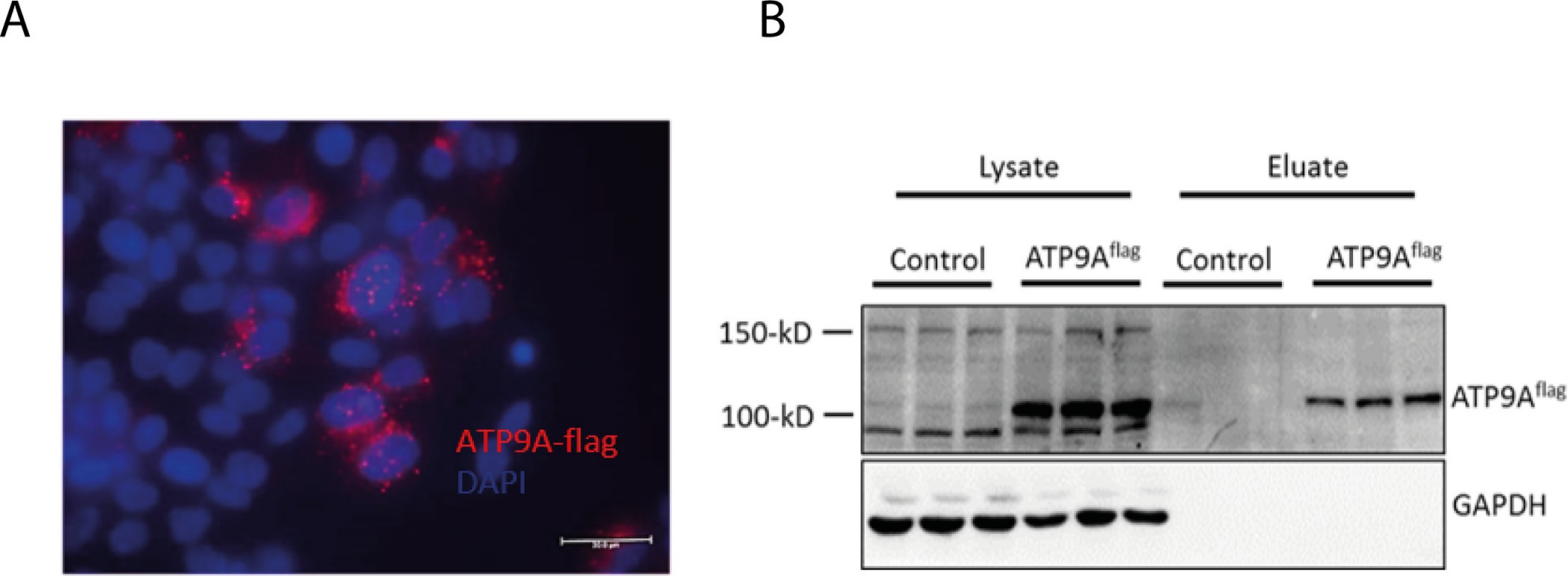
ATP9A localizes to intracellular vesicles and to the plasma membrane. Immunostaining and surface biotinylation was performed in ATP9A-flag overexpressing HepG2 cells to study the localization of ATP9A. (A) Immunostaining of ATP9A-flag (red) in HepG2. Nuclear DAPI staining in blue. (B) Surface biotinylation of ATP9A-flag in over-expressing HepG2 cells. Immunoblot showing ATP9A and GAPDH in the total lysate and eluate fraction.

To further investigate the role of ATP9A in the release of EVs, the cellular localization of ATP9A in HepG2 cells was studied. Cells over-expressing flag-tagged ATP9A (hereafter termed ATP9A^flag^) were generated, and the localization of ATP9A^flag^ was investigated. Immunofluorescent detection revealed that ATP9A^flag^ is predominantly present in intracellular vesicles (Fig 3A). Although no ATP9A^flag^ was detectable at the cell membrane, surface biotinylation did reveal its presence at the plasma membrane of HepG2 cells (Fig 3B). Quantification of protein bands in the eluate and total lysate revealed that the majority of ATP9A^flag^ localized to intracellular compartments, while 10 to 15% of ATP9A^flag^ was biotinylated and thus present in the plasma membrane. Overall these data clarified that ATP9A is predominantly present in intracellular compartments and is transiently present at the plasma membrane.

### ATP9A depleted cells release ceramide-dependent exosomes

**Fig 4:**
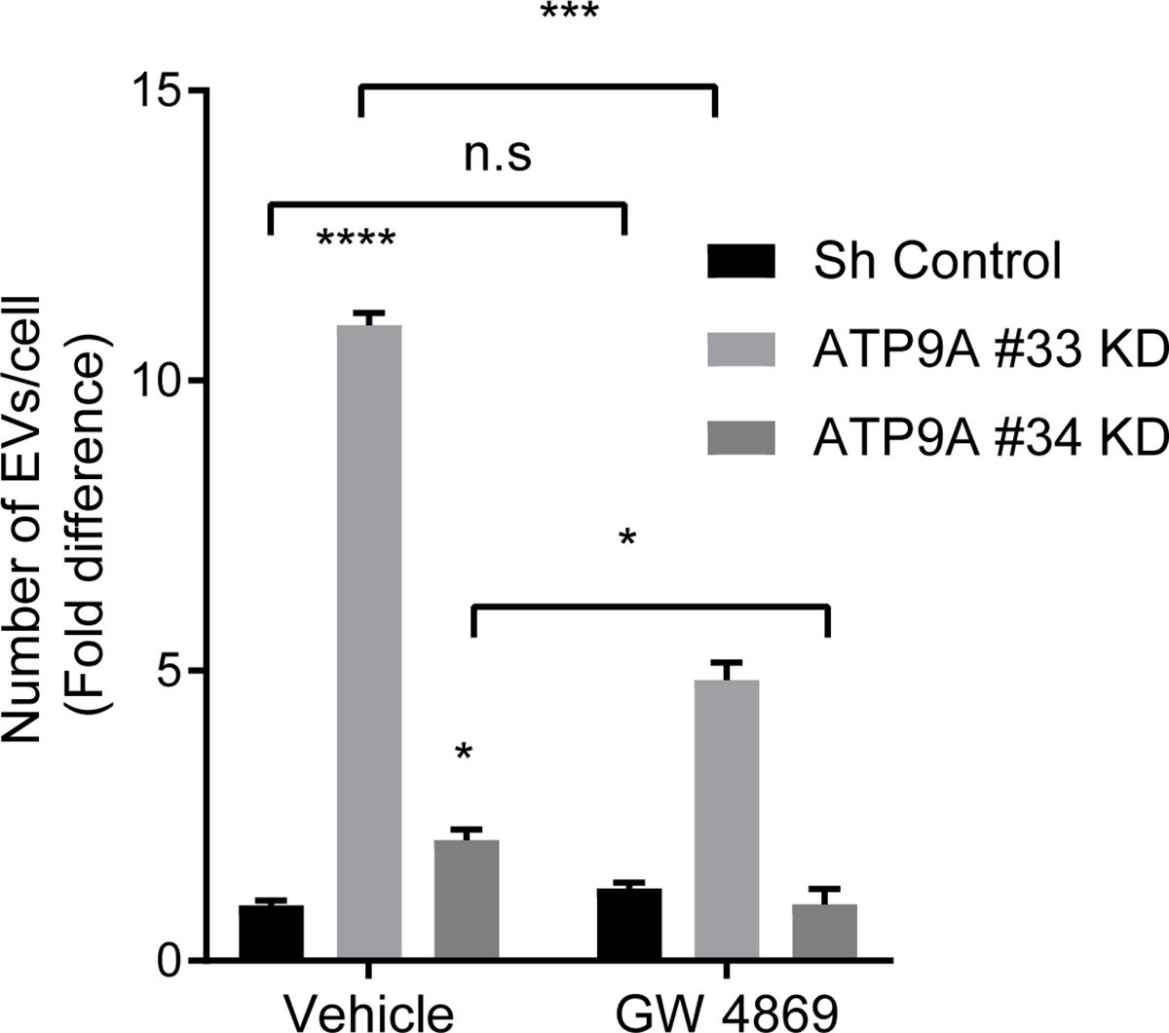
Ceramide dependent release of EVs in ATP9A knock-down cells. ATP9A knock-down and sh-control cells were incubated for 66 hours with GW4869 (10μM) or vehicle (1% DMSO). The mean of excreted EVs/cell of a representative of 3 independent experiments all performed in triplo (n = 9) ± SD is shown. Statistical significance was tested by two-way ANOVA with Tukey’s correction for multiple testing: ***p<0.0005 and n.s p≥0.05.+ represents the nonsignificant difference.

Since our data revealed that ATP9A is present both in intracellular vesicles and at the plasma membrane, its depletion may affect either EV release from the plasma membrane (i.e. ectosomes) or exosome release via multivesicular endosome (MVE) fusion with the plasma membrane. To investigate which pathway was affected by ATP9A depletion, we used the established neutral sphingomyelinase (nSMase) inhibitor GW4869. nSMases catalyze the conversion of sphingomyelin to ceramide, that is essential for the biogenesis of the MVE. It is well established that inhibition of nSMases by GW4869 reduces exosome release [25, 26] but not ectosome release [27]. The effect of 10 μM GW4869 on EV release in control and *ATP9A* depleted HepG2 was investigated and clearly showed that GW4869 significantly reduced the total number of EVs released by ATP9A depleted cells (Fig 4). This concentration GW4869 was not toxic to the cells as cell viability after a 66 hour incubation period was not affected (S2 Fig). These data indicate that the enhanced release of EVs from ATP9A depleted cells is predominantly due to an increased excretion of exosomes.

### ATP9A depletion interferes with endocytosis, apoptosis, and proliferation

**Fig 5:**
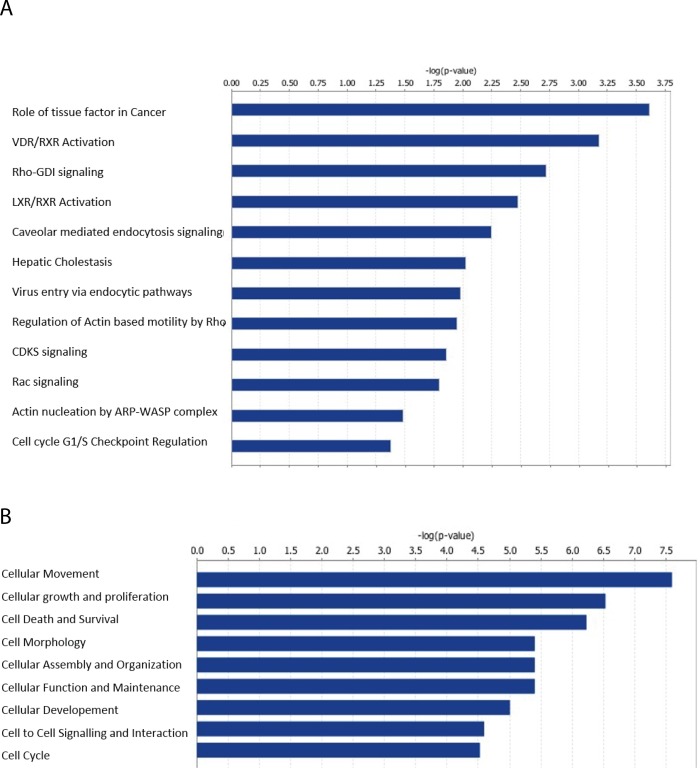
ATP9A knock-down in HepG2 cells affects endocytic pathways. RNA was isolated from sh-control and ATP9A knock-down HepG2 cells at 72 hours after transduction and used for microarray analysis. (A) Ingenuity pathway analysis of significantly up-regulated and down-regulated genes enriched for multiple pathways related to endocytosis. (B) Gene Ontology (GO) molecular function analysis of significantly altered genes enriched for the indicated pathways affecting cell migration, cell growth and proliferation, and cell death and survival. Pathways are arranged in order of their significance [-log(p-value)].

**Fig 6:**
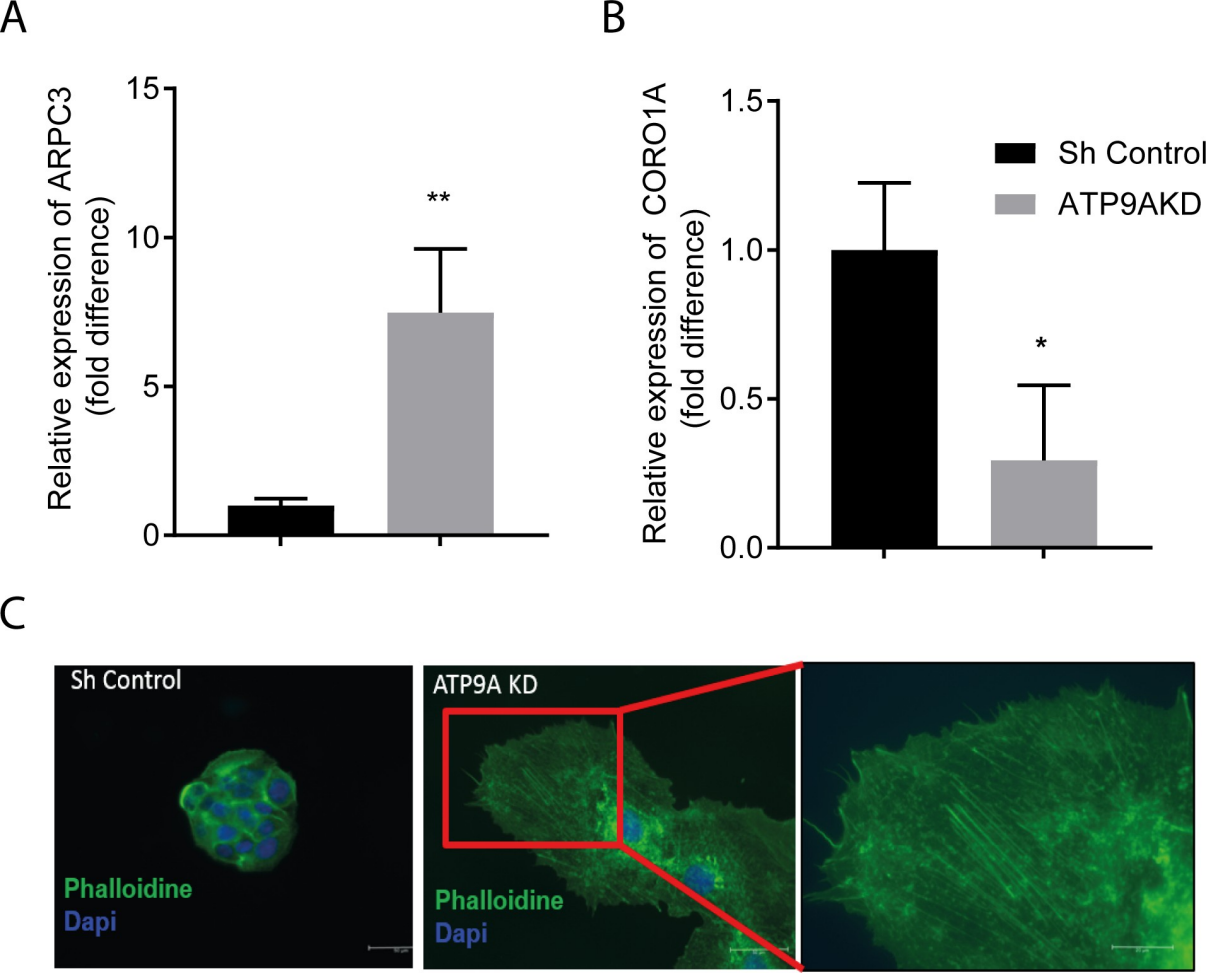
ATP9A knock-down affects *ARPC3* and *CORO1A* expression and leads to changes in the actin cytoskeleton. (A) Relative mRNA expression of *ARPC3* in ATP9A knock-down cells compared to sh-control cells. (B) Relative mRNA expression of *CORO1A* in ATP9A knock-down cells compared to sh-control cells. (C) Phalloidin staining for actin filaments (green) and DAPI staining for nuclei (blue) in control and ATP9A knock-down cells. Statistical significance was tested by a student’s t-test: *p<0.05, **p<0.005.

To clarify the molecular mechanism of the enhanced EV release and the lethality caused by ATP9A depletion, a gene expression microarray was performed in shControl and *ATP9A* knock-down cells 72 hour post-transduction. The microarray data are available from the NCBI gene expression Database with the accesion number GSE123399. Comparison of two different *ATP9A* knock-down HepG2 lines with the shControl revealed a significant >2 fold increase in mRNA expression of 75 genes and a significant >2 fold reduction of 50 genes (S1 and S2 Tables). ATP9A depletion altered the levels of proteins in various pathways, including endocytosis, actin nucleation and actin-based motility (Fig 5A). Based on a GO term analysis, the cellular functions most affected by ATP9A depletion include cell motility and morphology, cell growth and proliferation, and cell death and survival (Fig 5B). The efficient 2.7-fold downregulation of *ATP9A* mRNA in the microarray underscored the efficiency of both short hairpin constructs (S1 Table). Coronin 1A (*CORO1A*) was the most significantly down-regulated gene (2-fold) in *ATP9A* depleted cells (S1 Table), while actin-related protein 2/3 complex subunit 3 (*ARPC3*) was among the strongest upregulated genes with a 3.5-fold over-expression upon ATP9A knock-down (S2 Table). Upon further validation with RT-qPCR, *ARPC3* was confirmed to be upregulated and *CORO1A* was confirmed to be downregulated in *ATP9A* depleted cells (Fig 6A and 6B). Intriguingly, both genes are involved in the regulation of actin polymerization. To assess defects in the actin cytoskeleton, actin filaments in *ATP9A* knock-down and control cells were stained with phalloidin 5 days after the transduction. In contrast to control HepG2 cells, which are small and typically grow in small islands with smooth cell contacts, *ATP9A* knock-down cells were highly expanded with long actin filaments and protrusions from the plasma membrane (Fig 6C), indicating that ATP9A depletion affects HepG2 cell morphology possibly caused by extensive cytoskeletal re-arrangements.

## ATP9A has no detectable flippase activity towards NBD-labeled phospholipids

**Fig 7:**
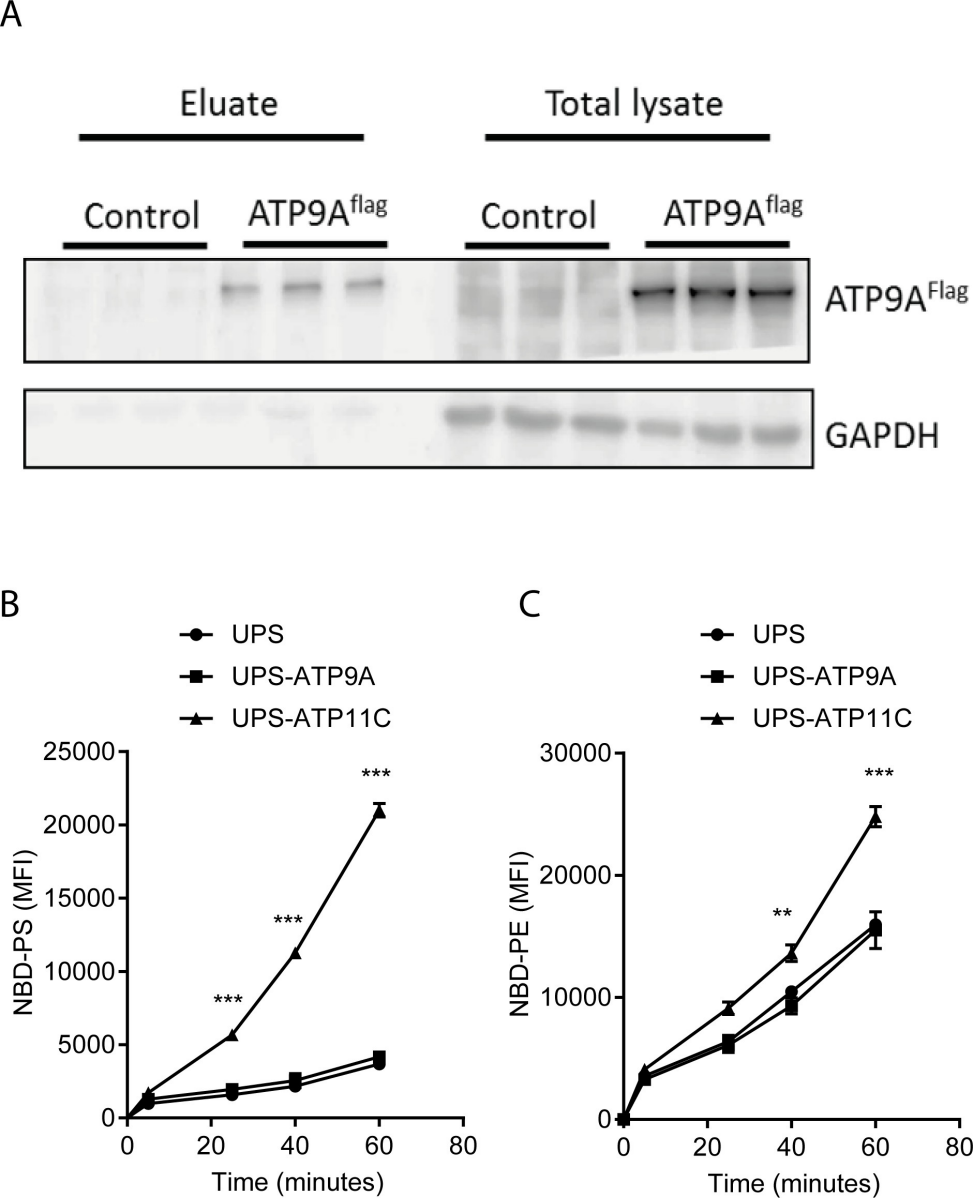
ATP9A does not transport NBD-labeled phospholipids. (A) UPS-1 cells overexpressing ATP9A-flag (UPS-ATP9A^flag^) and control cells were incubated with 1 mg/ml sulfur-NH-ss-biotin for 1 hour. Biotinylated proteins were isolated and the presence of ATP9A-flag and GAPDH was analyzed by Western blot. (B and C) UPS-ATP9A^flag^ cells were incubated with NBD-PS and NBD-PE respectively for 15, 30 and 60 minutes and internalization of NBD–phospholipids was analyzed by FACS. Mean fluorescent intensity of (B) NBD-PS, (C) NBD-PE was plotted over 60 minutes. Statistical significance was determined by one-way ANOVA with Bonferroni correction for multiple testing. *p<0.05,**p<0.005.

Given the mild increase in PS externalization in ATP9A depleted cells, we studied the phospholipid flippase activity of ATP9A. ATP9A^flag^ was over-expressed in Chinese Hamster Ovary-derived UPS-1 cells, which are knockout cells for the hamster P4-ATPase ATP11C [28]. Similar to HepG2 cells, surface biotinylation of UPS-ATP9A^flag^ cells showed that 10–15% of the ATP9A^flag^ pool localized to the plasma membrane in hamster cells (Fig 7A). Internalization of NBD-labeled phospholipid analogs NBD-PS and NBD-PE was studied in UPS-ATP9A^flag^ cells (Fig 7B–7D). ATP11C^flag^ over-expressing UPS-1 cells were included as a positive control for NBD-PS and NBD-PE internalization, as ATP11C is known to have flippase activity towards these two NBD-labeled phospholipids [29]. As expected, the internalization of NBD-PS and NBD-PE was significantly and time-dependently increased in ATP11C-overexpressing cells compared to control cells; however, ATP9A^flag^ cells showed no enhanced uptake of these phospholipid analogs (Fig 7B and 7C). These data indicate that ATP9A^flag^ does not show detectable flippase activity towards the tested NBD-labeled phospholipids.

## Discussion

Here we show that the P4-ATPase ATP9A plays an important role in the release of EVs from human cells. Depletion of ATP9A from cell lines of different origin, including HepG2, MCF-7 and THP-1, resulted in enhanced EV release, suggesting an universal role of ATP9A in this process. EVs play crucial roles in intercellular communication by delivering proteins, DNA, miRNA and mRNAs that regulate a wide range of processes, including blood clotting, angiogenesis, detoxification and immune responses. This requires tight regulation of EV release, however, the mechanisms hereof are poorly understood. Our data suggest that ATP9A inhibits the secretion of exosomes, as inhibition of neutral sphingomyelinase with GW4869 resulted in a normalization of EVs release in *ATP9A*-depleted HepG2 cells.

Our gene expression microarray analyses in *ATP9A*-depleted HepG2 cells revealed a strong induction of actin-related protein 2/3 complex subunit 3 (ARPC3) and a strong down-regulation of coronin 1A. Activation of ARP2/3 complex leads to actin nucleation and stabilization of the branched actin network. ARP2/3-mediated actin branching is negatively regulated by coronin, which results in de-branching [30–32]. Importantly, coronin 1A binds to ARPC2/3, thereby inhibiting its actin nucleation activity [33]. Sinha *et al*. recently reported dynamic regulation of exosome secretion by cortactin and coronin 1B, and showed that coronin 1B antagonized cortactin resulting in actin de-branching with a consequent reduction in exosome secretion[34]. Stabilized, branched actin structures are thought to serve as docking sites for MVEs to facilitate the release of exosomes [35]. Cortactin is an actin binding protein that interacts and activates the ARP2/3 complex. Cortactin was also identified as an important regulatory protein in exosome secretion as depletion and overexpression of cortactin resulted in reduced and increased exosome secretion, respectively [34, 36]. We speculate that the strongly enhanced EVs secretion in ATP9A-depleted cells is a combined consequence of reduced coronin 1A and induced ARP2/3 activity, which leads to more actin nucleation. This may also explain the long actin fibers observed in ATP9A-depleted HepG2 cells.

Our work indicates that ATP9A negatively controls exosome secretion, however, how ATP9A is involved herein remains to be determined. ATP9A is a member of a family of proteins that are implicated in the trafficking of proteins, more specifically in the initiation step of vesicle biogenesis [15, 18, 19]. Recently, Tanaka *et al*. [22] showed that ATP9A is indispensable for the endocytic recycling to the plasma membrane of transferrin receptor and glucose transporter 1 (GLUT1). One scenario may be that ATP9A mediates the endocytic recycling of proteins involved in the regulation of cytoskeletal re-arrangements, for instance at MVE docking sites. Rab27a plays an essential role at the plasma membrane and facilitates docking of MVEs [37], but also stabilizes MVE docking sites by inhibiting coronin function [34]. ATP9A may regulate Rab27a activity at the plasma membrane via endocytic recycling of this protein, however, such a role needs to be demonstrated. Alternatively, enhanced EVs secretion could be a secondary consequence of defects in other essential processes. Our pathway analysis revealed that cell proliferation, cell death and survival pathways were affected by ATP9A depletion. For instance, several genes involved in cell proliferation, such as cyclin dependent kinase inhibitor proteins (CDKN1A, CDKN2B) and B cell translocation gene 2 (BTG2)) were significantly upregulated, possibly leading to cell cycle arrest and premature cell death. Indeed, *ATP9A*-depleted HepG2 cells were undergoing massive cell death approximately two weeks after depletion of ATP9A. ATP9A belongs to subclass 2 in the P4-ATPase phylogenetic tree [18], members of which include the ATP9A ortholog TAT-5 (*C*. *elegans*) and Neo1 (*S*. *cerevisiae*). Deficiencies in these P4-ATPases are lethal, however, the molecular mechanisms underlying cellular lethality are not understood [20, 38]. Despite the lethality in HepG2 cells (this study), CRISPR-mediated knockout of ATP9A was not lethal in Hela cells, although no long-term culture data were presented [22]. ATP9A depletion is also not lethal in HEK293T, MCF-7 or in THP-1 cells (unpublished observation by us). This indicates that ATP9A depletion can be tolerated in some cell types, which may depend on the pathways in which the protein is involved.

A partial loss of plasma membrane asymmetry in ATP9A depleted HepG2 cells was evidenced by increased exposition of PS in the exoplasmic leaflet. In the yeast *Saccharomyces cerevisiae*, deficiency of the yeast ortholog of ATP9A, NEO1, results in loss of plasma membrane asymmetry by exposition of PS and PE [38]. TAT-5 deficiency in *C*. *elegans* also showed enhanced exposition of PE in the exoplasmic leaflet of the membrane, while no PS externalization was observed [20]. Although we did measure enhanced exposition of PS in ATP9A-depleted HepG2 cells, our attempts to measure exposition of PE were not successful. In addition, we could not detect any ATP9A-mediated transport activity for NBD-PE and NBD-PS, which may be due to low plasma membrane presence of ATP9A^flag^ (~10% of total), or that ATP9A transports endogenous PS and/or PE and has (too) low affinity for its NBD-labeled phospholipid analogs. Most of the ectopically-expressed ATP9A^flag^ was present in intracellular, possibly endosomal compartments. Two recent studies showed localization of ectopically-expressed ATP9A in early- and recycling endosomes and in the *trans*-Golgi network in HeLa cells [21, 22]. We find ATP9A in intracellular compartments, but also at the plasma membrane, which implies a transient presence of ATP9A in the plasma membrane and supports a role for ATP9A in endocytic recycling of plasma membrane proteins. We therefore cannot exclude the possibility that loss of membrane asymmetry in ATP9A-depleted cells is a secondary consequence of a general defect in the (endocytic) trafficking of other membrane flippases that maintain asymmetry. However, ATP9A depletion did not affect protein levels of ATP11C, a P4-ATPase that catalyzes PS flipping in the plasma membrane [24] (not shown). These observations suggest minor, if any redundancy in P4-ATPase-mediated maintenance of plasma membrane asymmetry in HepG2 cells. In conclusion, we have identified the P4-ATPase ATP9A as a negative regulator of exosome release.

## Materials and methods

### Cell culture

All the cell lines used in this study were obtained from ATCC. Human hepatocellular carcinoma (HepG2), human embryonic kidney (HEK293T) and Chinese Hamster ovary (CHO-K1) cells were cultured in DMEM with 10% Fetal bovine serum (FBS), 2 mM L-glutamine, 100 U/ml penicillin, and 100 U/ml streptomycin (all from Lonza, Verviers, Belgium). Human breast cancer (MCF-7) cells were cultured in DMEM with 7.5% FBS, 2 mM L-glutamine, 100 U/ml penicillin, and 100 U/ml streptomycin. Human monocytic leukemia cells (THP-1) were cultured in RPMI (Gibco,NewYork, USA) with 10% FBS, 2 mM L-glutamine, 100 U/ml penicillin, and 100 U/ml streptomycin. All cell lines were cultured at 37°C in a 5% CO_2_ humidified incubator.

GW4869 (Sigma Aldrich) was used to inhibit neutral sphingomyelinases. 5 μM Q-VD-OPh((3S)-5-(2,6-Difluorophenoxy)-3-[[(2S)-3-methyl-1-oxo-2-[(2-uinolinylcarbonyl)amino] butyl] amino]-4-oxo-pentanoic acid hydrate (Sigma Aldrich) was used to inhibit caspase 3/7 activity. Chenodeoxycholate (CDC) was used to induce caspase activity. Caspase-3/7 activity was determined by using the SensoLyte Homogeneous AMC Caspase-3/7 Assay kit (Anaspec Inc, San Jose, CA) according to the manufacturer’s protocol.

### Lentivirus production and lentiviral transduction of cells

Plasmids containing shRNA to *ATP9A* (TRCN0000050733, TRCN0000050734, TRCN0000050735, TRCN0000050736, TRCN0000050737, MISSION shRNA Library; Sigma/Aldrich) were produced in *E*. *coli*. The self-inactivating lentiviral vectors delivering shRNA sequences were produced in 293T cells as described previously [39]. Briefly, expression vectors for HIV Gag/Pol, HIV Rev, VSVg and the viral transfer vector were co-transfected using PEI into HEK293T cells. The next day the culture medium replaced and 24 hours later virus containing supernatants were collected and passed through a 0.45 μm filter.

Cells were incubated with virus-containing supernatants supplemented with 10 μg/ml diethylaminoethyl-dextran for 4 hours after which the medium was refreshed. Two days post-transduction, cells were selected on 2 μg/ml puromycin. Knock-down of *ATP9A* was accessed in every experiment by qRT-PCR. Among the five shRNAs tested TRCN0000050733 (shATP9A #33) and TRCN0000050734 (shATP9A #34) provided the most efficient knock-down of *ATP9A* and were used throughout this study.

### Quantification of EV in culture medium

48 hours after lentiviral transduction, medium was replaced with serum free medium or EV-depleted medium generated by centrifugation of FBS-containing medium at 10,621x g for 16 hours. Next day, the medium was replaced again with serum free or EV-depleted medium (w/o 10μm GW4869), and 66 hours later the medium was harvested and centrifuged at 200x g for 5 minutes to remove the dead cells, followed by a centrifugation at 2,000xg for 20 min to pellet cell debris larger vesicles and cell particles. The supernatant was diluted 20 times with 0.2 μm filtered phosphate buffer saline (PBS) to quantify EVs by nanoparticle tracking analysis (NTA) using a NanoSight as described [23].

### RNA isolation and quantitative RT-PCR

Total RNA was isolated 48 hours post transduction using TriReagent (Sigma) according to the manufacturer’s protocol. cDNA was synthesized using 2 μg of total RNA with oligo dT and random hexamer primers and RevertAid reverse transcriptase (Thermo Scientific). RT-PCR measurements were performed on a Lightcycler 480 (Roche) with Sensifast Syber no-rox (Bioline). The primers for *ATP9A*: forward 5′-AGCAAACGTATGGGCATCAT-3′; reverse 5′-TGCTCCTCTGCAAGAGACTTC-3′. Hypoxanthine-guanine phosphoribosyl-transferase (*HPRT*) forward 5′-CATTATGCTGAGGATTTGGAAAGG-3′; reverse 5′-CTTGAGCACACAGAGGGCTACA-3′- and *36B4* with the forward primer 5′-TCATCAACGGGTACAAACGA-3′ and reverse primer 5′- GCCTTGACCTTTTCAGCAAG-3′ were used as reference genes. *ATP9A* transcript levels were calculated by LC Conversion and Linreg PCR program and normalized with the geometric mean of two reference genes [40].

### Gene expression microarray (Illumina)

72 hours after transduction with shATP9A lentiviral constructs, cells were lysed and RNA was isolated using the isolate II RNA mini kit (Bioline). 150 ng of RNA was labeled with cRNA labeling kit for Illumina BeadArray (Ambion) and hybridized with Ref8v3 BeadArray (Illumina). Arrays were scanned on a BeadArray 500GX scanner. Using Genome Studio software (Illumina), arrays were normalized using quantile normalization, and the background was subtracted. All genes that had negative values were removed from the analysis. Differentially expressed genes were extracted using remote analysis computation for gene expression data. Differentially expressed genes had a p-value <0.05 (analysis of variance), followed by a false discovery rate (FDR) post analysis correction for multiple comparisons.

### Phosphatidylserine exposure

Cells were harvested 116 hours after lentiviral transduction and washed with 1 ml of ice-cold PBS, and labeled with Lactadherin-FITC (BD Bioscience) diluted 1:100 in PBS for 20 minutes at 37°C. After three washes with ice cold PBS, the cells were taken up in ice cold PBS and analyzed by FACS for presence of FITC.

### Generation of HepG2 cells over-expressing ATP9A^flag^, and RAB5, RAB7 or RAB11

cDNA encoding ATP9A was obtained from the PlasmID Repository/DF/HCC DNA Resource Core (http://plasmid.med.harvard.edu) and inserted into a lentiviral vector behind a human PGK promoter. A FLAG-tag was introduced at the carboxy terminus by PCR using Phusion polymerase (Thermo scientific); forward-oligo5′-cattagctacgaccggtatggactgcagcctcgtgcggacg-3′; reverse oligo 5′-ggctggtctagactaGACTACAAGGACGACGATGACAAGgaaactcaggctgctggaag-3′ (capital letters indicate the FLAG sequence).

### Surface biotinylation and Western blot analysis

HepG2 and UPS-1 cells overexpressing ATP9A^flag^ were washed 3x with ice-cold phosphate buffered saline (pH 8.2) containing 1 mM MgCl_2_ / 0.1 mM CaCl_2_ (PBS-CM) and incubated with 1 mg/ml sulfo-NH-SS-biotin (Thermo Scientific) in ice-cold PBS-CM for 60 min while gently shaken. To quench the non-bound biotin, cells were washed twice for 5 min with ice-cold PBS-CM containing 100 mM glycine and once more with ice-cold PBS-CM. Cells were lysed for 30 min on ice in RIPA buffer (50 mM Tris-HCl pH 8.0, 150 mM NaCl, 1% NP-40, 0.5% Na-deoxycholate, 0.1% SDS) containing protease inhibitor cocktail (Roche). Biotinylated protein was precipitated with high-capacity neutravidin agarose resin (Thermo Scientific) for 2 h at 4°C. Beads were washed four times with RIPA buffer and in between washes spun down for 1 min at 450 x g. Biotinylated protein was eluted with DTT- and SDS-containing sample buffer at room temperature. Total lysates and eluates were analyzed by SDS-PAGE and Western blotting. The PVDF membrane was blocked in 5% milk (Nutricia Profitar-plus) in PBS/0.05% Tween-20 and incubated for 1 hour at room temperature with anti-flag antibody (Sigma, F1804) 1:1000 in 1% milk in PBS/0.05% Tween-20. Immune complexes were visualized with peroxidase-conjugated goat-anti-mouse (Bio-Rad) and developed with homemade enhanced chemiluminescence reagents (100 mM Tris–HCl, pH 8.5, 1.25 mM luminol, 0.2 mM p-coumarin and freshly added 3 mM hydrogen peroxide). Signals were detected in ImageQuant™ LAS 4000 (GE Healthcare).

### Immunostaining

ATP9A knock-down HepG2 cells, ATP9A^flag^ tagged with GFP over expressing HepG2 cells were grown on glass coverslips. Cells were washed with PBS and fixed with 2% paraformaldehyde (PFA) for 15 minutes followed by permeabilization with 0.1% Triton-X 100 in PBS. ATP9A knock-down cells were incubated with 1:100 phalloidin Alexa 488 (Molecular Probe, A12379) or phalloidin Alexa 594 (Molecular Probe, A12384) for 1 hour at room temperature. ATP9A^flag^ cells were incubated with FITC labeled anti-flag mouse monoclonal antibody (Sigma, F1804) in 1:100 in 0.1% triton X-100 for 1 hour followed by 1 hour incubation with goat anti-mouse 1:2000 in 0.1% triton X-100.Then, the cells were washed with 0.1% Triton X-100 3 times for 5 minutes each and fixed with vectashield containing DAPI.

### NBD-phospholipid transport assay

Uptake of fluorescently-labeled phospholipid analogs 16:0–06:0 phosphatidylserine, ethanolamine and choline (1-palmitoyl-2-{6-[(7-nitro-2-1,3-benzoxadiazol-4-yl)amino]hexanoyl}knock-down-sn-glycero-3-phosphoserine, phosphoethanolamine and phosphocholine) (Avanti polar-lipids, Inc., Alabaster, USA) was studied as described [29]. Briefly, NBD-PL stock in chloroform/methanol (3:2) was transferred to a glass tube and dried under a stream of nitrogen. The lipid film was dissolved in 96% ethanol using glass beads. Subsequently, Hanks Balanced Salt Solution without phenol red (Cambrex, Verviers, Belgium) supplemented with 20 mM Hepes, pH 7.4 (HBSS/H) was added while vortexing to a final concentration of 2 **μ**M NBD-PL in HBSS/H. ATP9A and ATP11C over-expressing Chinese hamster ovary (CHO)-K1-derived UPS-1 (uptake of fluorescent PS analogs) cells, which are defective in PS internalization [41], were trypsinized and equilibrated for 15 minutes in HBSS/H. Cells were incubated with NBD-PS, NBD-PE and NBD-PC at 10°C and samples were taken at indicated time points. After back extraction of exoplasmic leaflet-associated NBD-PL with 5% fatty acid-free bovine serum albumine in HBSS, cellular fluorescence was quantified by flow cytometry using a LSR Fortessa (BD Biosciences).

### Statistics

Data expressed as mean ± standard deviation (SD) or mean ± standard error of mean (SEM) are plotted with Prism 5 (Graphpad software). Statistical significance was determined by Student's t-test or one-way ANOVA with Dunnett’s or Bonferroni correction for multiple comparison or two way ANOVA with Tukey correction for multiple comparison as indicated in the legend of the figures.

## Supporting information

S1 Fig Knock-down of ATP9A in THP-1 and MCF-7 cells increases EV release. MCF-7 and THP-1 cells were transduced with lentiviral vectors encoding shATP9A #33, #34 or sh-control. 116 hours after RNA was isolated from the cells and medium was harvested to isolate EVs. (A) Relative mRNA expression of *ATP9A* to reference gene *HRPT* in THP-1 cells. (B) Relative mRNA expression of *ATP9A* to reference gene *HRPT* in MCF-7 cells. (C) Number of EVs per cell in THP-1 cells. (D) Number of EVs per cell in MCF-7 cells. Values were plotted as mean ± SD. Student’s t-test or one-way ANOVA was done to test the statistical significance: *p< 0.05, **p<0.005.
(TIF)

S2 Fig Cell survival is unaffected with 10μM GW4869. HepG2 cells were transduced with shATP9A #33, #34 or sh control to deplete ATP9A and incubated with 10μM GW4869 or vehicle (DMSO) for 66-hours. Cell survival was unaffected with 10μM GW4869. HepG2 cells were transduced with shATP9A #33, #34 or sh control to deplete ATP9A and incubated with 10μM GW4869 for 66-hours or vehicle (DMSO). WST assay was performed after seeding 10,000 parental, Sh control, ATP9A#33 KD and ATP9A #34 KD HepG2 cells/well in a 96 well plate. Student ‘t’ test was done to test the statistical significance, n.s, not significant. WST assay was performed after seeding 10,000 parental, Sh control, ATP9A#33 KD and ATP9A #34 KD HepG2 cells/well in a 96 well plate. Student ‘t’ test was done to test the statistical significance, n.s, not significant.
(TIF)

S1 Table List of down regulated genes in ATP9A knock-down cells. (DOCX)

S2 Table List of upregulated genes in ATP9A knock-down HepG2 cells. (DOCX)
