# Supplementary figures and images for: The P4-ATPase ATP9A is a novel determinant of exosome release
Source: PLoS One. 2019 Apr 4;14(4):e0213069. doi: 10.1371/journal.pone.0213069 (PMC6448858; doi:10.1371/journal.pone.0213069)

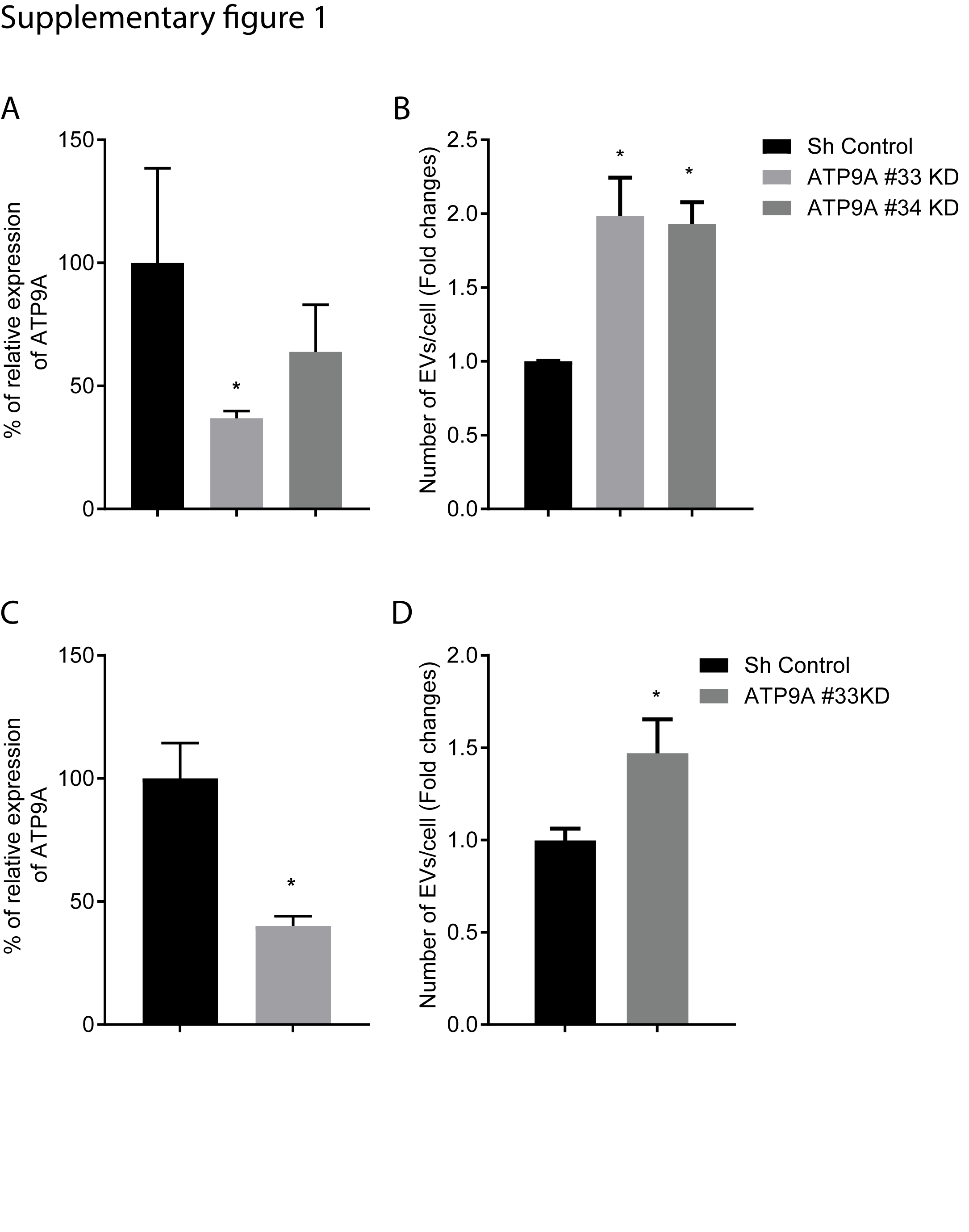

Supplement: S1 Fig — MCF-7 and THP-1 cells were transduced with lentiviral vectors encoding shATP9A #33, #34 or sh-control. 116 hours after RNA was isolated from the cells and medium was harvested to isolate EVs. (A) Relative mRNA expression of ATP9A to reference gene HRPT in THP-1 cells. (B) Relative mRNA expression of ATP9A to reference gene HRPT in MCF-7 cells. (C) Number of EVs per cell in THP-1 cells. (D) Number of EVs per cell in MCF-7 cells. Values were plotted as mean ± SD. Student’s t-test or one-way ANOVA was done to test the statistical significance: *p< 0.05, **p<0.005. (TIF) [file pone.0213069.s001.tif]

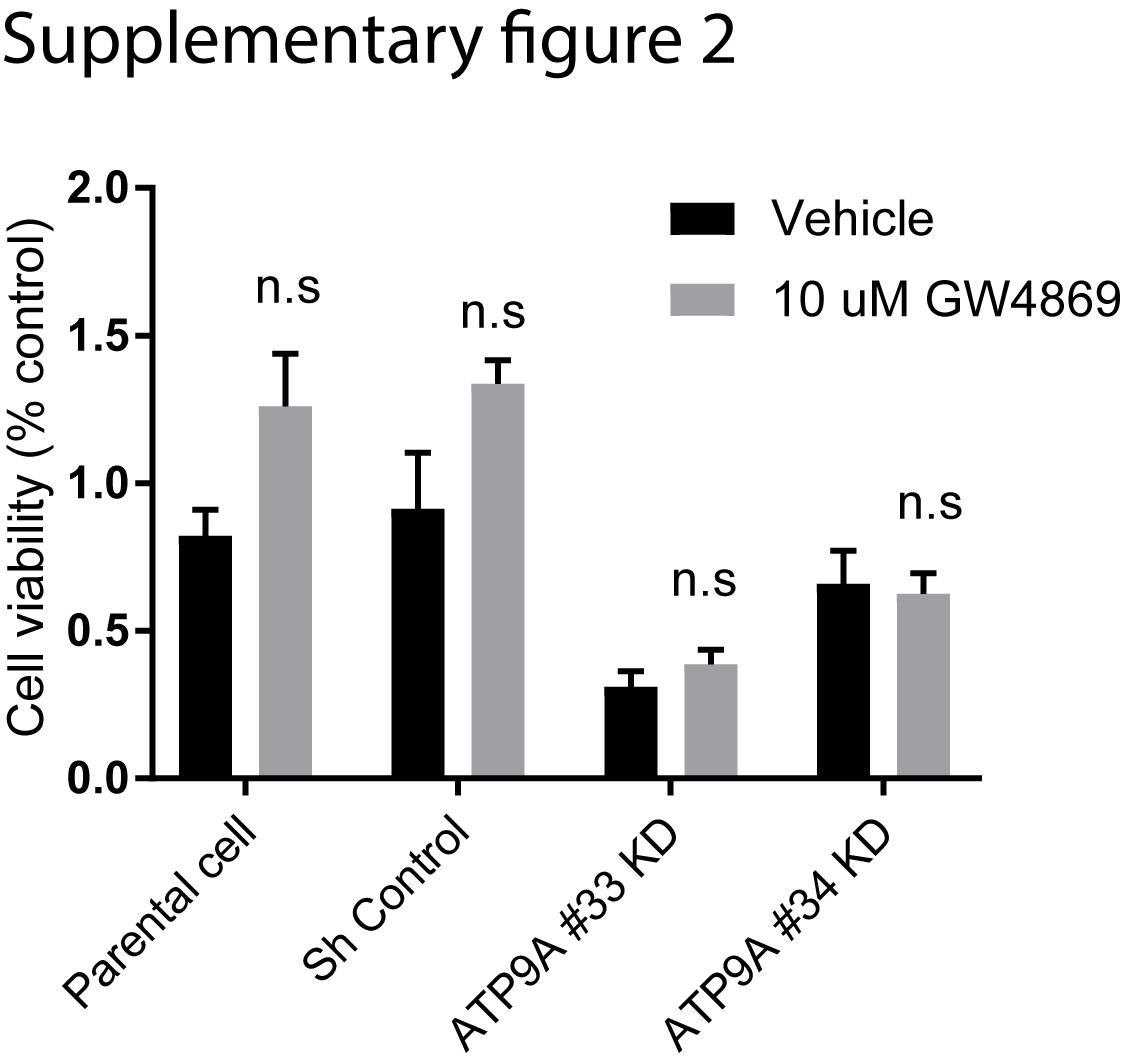

Supplement: S2 Fig — HepG2 cells were transduced with shATP9A #33, #34 or sh control to deplete ATP9A and incubated with 10μM GW4869 or vehicle (DMSO) for 66-hours. Cell survival was unaffected with 10μM GW4869. HepG2 cells were transduced with shATP9A #33, #34 or sh control to deplete ATP9A and incubated with 10μM GW4869 for 66-hours or vehicle (DMSO). WST assay was performed after seeding 10,000 parental, Sh control, ATP9A#33 KD and ATP9A #34 KD HepG2 cells/well in a 96 well plate. Student ‘t’ test was done to test the statistical significance, n.s, not significant. WST assay was performed after seeding 10,000 parental, Sh control, ATP9A#33 KD and ATP9A #34 KD HepG2 cells/well in a 96 well plate. Student ‘t’ test was done to test the statistical significance, n.s, not significant. (TIF) [file pone.0213069.s002.tif]
