# Supplementary material for: The P4-ATPase ATP9A is a novel determinant of exosome release
Source: PLoS One. 2019 Apr 4;14(4):e0213069. doi: 10.1371/journal.pone.0213069 (PMC6448858; doi:10.1371/journal.pone.0213069)
Supplement: S1 Table — (DOCX) [file pone.0213069.s003.docx]

Supplementary table-1: List of down regulated genes in ATP9A knock-down cells

| No | Gene symbol | Gene name | Fold differences | P value |
| --- | --- | --- | --- | --- |
| 1 | CORO1A | coronin, actin binding protein, 1A | -2 | 3,46E-05 |
| 2 | ATP9A | ATPase, class II, type 9A | -2,72 | 3,45E-04 |
| 3 | ICK | intestinal cell (MAK-like) kinase | -3,11 | 6,24E-04 |
| 4 | NFIX | nuclear factor I/X (CCAAT-binding transcription factor) | -2,64 | 1,05E-03 |
| 5 | PLCXD1 | phosphatidylinositol-specific phospholipase C, X domain 1 | -2,11 | 1,92E-03 |
| 6 | MAPT | microtubule-associated protein tau | -2,03 | 2,26E-03 |
| 7 | DLX1 | distal-less homeobox 1 | -2,07 | 2,90E-03 |
| 8 | IL17D | interleukin 17D | -2 | 3,04E-03 |
| 9 | RBP4 | retinol binding protein 4, plasma | -2,12 | 3,05E-03 |
| 10 | OAF |  | -2,14 | 3,19E-03 |
| 11 | GPAM | Glycerol 3 phosphate Acyl transferase mitochondrial | -2,73 | 3,44E-03 |
| 12 | SLC22A7 | Solute carrier family 22 member 7 | -2,41 | 3,49E-03 |
| 13 | CEBPA | CCAAT/enhancer binding protein (C/EBP), alpha | -2,78 | 4,16E-03 |
| 14 | PAQR8 | progestin and adipoQ receptor family member VIII | -2,39 | 4,32E-03 |
| 15 | NR1H4 | nuclear receptor subfamily 1, group H, member 4 | -2,62 | 4,67E-03 |
| 16 | ATP6V0E2 | ATPase, H+ transporting V0 subunit e2 | -2,23 | 4,72E-03 |
| 17 | TMEM41A | transmembrane protein 41A | -2,09 | 5,20E-03 |
| 18 | SLC25A1 | solute carrier family 25 | -2,47 | 5,53E-03 |
| 19 | CPN2 | carboxypeptidase N, polypeptide 2 | -2,41 | 5,78E-03 |
| 20 | SLC6A10P | solute carrier family 6 MEMBER 10 | -2,17 | 5,84E-03 |
| 21 | CERK | ceramide kinase | -2,04 | 6,26E-03 |
| 22 | HOXD1 | homeobox D1 | -3,08 | 8,96E-03 |
| 23 | THG1L | tRNA-histidine guanylyltransferase 1-like | -2,05 | 9,29E-03 |
| 24 | GNG7 | guanine nucleotide binding protein (G protein), gamma 7 | -2,13 | 9,82E-03 |
| 25 | TNFSF4 | tumor necrosis factor (ligand) superfamily, member 4 | -2,25 | 9,83E-03 |
| 26 | AIF1L | allograft inflammatory factor 1-like | -2,28 | 9,84E-03 |
| 27 | PANX2 | pannexin 2 | -2,27 | 0,011 |
| 28 | HSD17B2 | hydroxysteroid (17-beta) dehydrogenase 2 | -2,16 | 0,011 |
| 29 | ERBB3 | erb-b2 receptor tyrosine kinase 3 | -2,14 | 0,015 |
| 30 | ENPP3 | ectonucleotide pyrophosphatase/phosphodiesterase 3 | -2,03 | 0,018 |
| 31 | LOC401720 |  | -2,28 | 0,019 |
| 32 | LHPP | phospholysine phosphohistidine inorganic pyrophosphate phosphatase | -2,08 | 0,02 |
| 33 | FAM83D | family with sequence similarity 83, member D | -2,14 | 0,02 |
| 34 | C9orf58 |  | -2,21 | 0,02 |
| 35 | LOC100134081 | allograft inflammatory factor 1-like | -2,15 | 0,024 |
| 36 | KANK4 | KN motif and ankyrin repeat domains 4 | -2,74 | 0,024 |
| 37 | C2orf82 |  | -2,04 | 0,025 |
| 38 | PNPLA7 | patatin-like phospholipase domain containing 7 | -2,04 | 0,025 |
| 39 | HEY1 | hes-related family bHLH transcription factor with YRPW motif 1 | -2,31 | 0,027 |
| 40 | C9orf140 |  | -2,16 | 0,029 |
| 41 | PCSK9 | proprotein convertase subtilisin/kexin type 9 | -2,97 | 0,029 |
| 42 | KLHL14 | kelch-like family member 14 | -2,37 | 0,03 |
| 43 | HADH | hydroxyacyl-CoA dehydrogenase | -2,19 | 0,033 |
| 44 | S100A9 | S100 calcium binding protein A9 | -2,97 | 0,036 |
| 45 | SP5 | Sp5 transcription factor | -2,04 | 0,036 |
| 46 | MACROD1 | MACRO domain containing | -2,09 | 0,036 |
| 47 | ANKRD38 | KN motif and ankyrin repeat domains 4 | -3,08 | 0,039 |
| 48 | PRELID1 | PRELI domain containing 1 | -2,05 | 0,043 |
| 49 | LOC100132863 | high mobility group box 1 pseudogene 37 | -2,1 | 0,044 |
| 50 | CENPM | centromere protein M | -2,05 | 0,047 |

Supplementary table-1: **:** List of genes that are significantly ≥2 fold downregulated in *ATP9A* depleted HepG2 cells as compared to controls. HepG2 cells were transduced with the shATP9A #33, #34 or sh-control. 72 hours after transduction, cells were harvested for RNA isolation. RNA from ATP9A knock-down and control cells was labeled with cRNA labeling kit for Illumina BeadArrays (Ambion) and hybridized with Ref8v3. Genes are presented in the order of their significance.
