# Supplementary material for: The P4-ATPase ATP9A is a novel determinant of exosome release
Source: PLoS One. 2019 Apr 4;14(4):e0213069. doi: 10.1371/journal.pone.0213069 (PMC6448858; doi:10.1371/journal.pone.0213069)
Supplement: S2 Table — (DOCX) [file pone.0213069.s004.docx]

Supplementary Table-2: List of upregulated genes in ATP9A knock-down HepG2 cells

| No | Gene symbol | Gene name | Fold differences | P value |
| --- | --- | --- | --- | --- |
| 1 | LOC441131 | Similar to Actin related protein 2 | 4,11 | 5,38E-05 |
| 2 | LOC644863 | signal recognition particle 14kDa | 2,21 | 5,54E-05 |
| 3 | ARPC3 | Actin related protein 3 | 3,5 | 5,89E-05 |
| 4 | CCDC90B | coiled-coil domain containing 90B | 2,57 | 7,55E-05 |
| 5 | LOC442609 | Similar to speedy homolog A, transcript variant 7 | 2,82 | 1,39E-04 |
| 6 | PEX11B | peroxisomal biogenesis factor 11 beta | 2,43 | 1,57E-04 |
| 7 | LOC729841 |  | 4,05 | 2,36E-04 |
| 8 | ITGA6 | integrin, alpha 6 | 2,04 | 2,84E-04 |
| 9 | SRP14 | signal recognition particle 14 | 2,13 | 4,34E-04 |
| 10 | HIST1H2BJ | histone cluster 1 | 2,18 | 1,35E-03 |
| 11 | SRP14P1 | signal recognition particle 14 pseudogene 1 | 2,12 | 2,02E-03 |
| 12 | SKA2 | spindle and kinetochore associated complex subunit 2 | 2,18 | 2,08E-03 |
| 13 | NPEPPS | aminopeptidase puromycin sensitive | 2,33 | 2,31E-03 |
| 14 | CYR61 | cysteine-rich, angiogenic inducer, 61 | 3,75 | 2,39E-03 |
| 15 | GCNT2 | glucosaminyl (N-acetyl) transferase 2 | 2,49 | 2,39E-03 |
| 16 | KIAA1324 | KIAA1324 | 2,79 | 2,72E-03 |
| 17 | SLC16A4 | solute carrier family 16, member 4 | 2,19 | 2,73E-03 |
| 18 | CCNYL1 | cyclin Y-like 1 | 2,14 | 3,05E-03 |
| 19 | COX7C | cytochrome c oxidase subunit VIIc | 2,39 | 3,36E-03 |
| 20 | TNFRSF10D | tumor necrosis factor receptor superfamily, member 10d | 2,33 | 3,44E-03 |
| 21 | TATDN3 | TatD DNase domain containing 3 | 2,18 | 3,65E-03 |
| 22 | ACOT9 | acyl-CoA thioesterase 9 | 2,24 | 3,78E-03 |
| 23 | TP53I3 | tumor protein p53 inducible protein 3 | 2,4 | 4,74E-03 |
| 24 | LOC100216001 | long intergenic non-protein coding RNA 704 | 2,61 | 5,07E-03 |
| 25 | SERPINE1 | serpin peptidase inhibitor, clade E member 1 | 2,53 | 5,33E-03 |
| 26 | F2RL1 | coagulation factor II (thrombin) receptor-like 1 | 2,41 | 5,36E-03 |
| 27 | LOC647859 |  | 2,02 | 5,42E-03 |
| 28 | BIN1 | bridging integrator 1 | 2,01 | 5,58E-03 |
| 29 | SH3RF1 | SH3 domain containing ring finger 1 | 2,01 | 5,65E-03 |
| 30 | EIF4E2 | ukaryotic translation initiation factor 4E family member 2 | 3,04 | 6,69E-03 |
| 31 | BTG2 | BTG family, member 2 | 4,45 | 6,76E-03 |
| 32 | TIMP2 | TIMP metallopeptidase inhibitor 2 | 2,57 | 6,83E-03 |
| 33 | ARHGDIB | Rho GDP dissociation inhibitor (GDI) beta | 2,65 | 8,13E-03 |
| 34 | PEAR1 | platelet endothelial aggregation receptor 1 | 2,21 | 8,17E-03 |
| 35 | ITGA3 | integrin, alpha 3 | 2,55 | 8,45E-03 |
| 36 | ANGPTL4 | angiopoietin-like 4 | 4,68 | 8,50E-03 |
| 37 | SDC4 | syndecan 4 | 3,75 | 8,92E-03 |
| 38 | DCP2 | decapping mRNA 2 | 2,24 | 0,01 |
| 39 | CMTM3 | CKLF-like MARVEL transmembrane domain containing 3 | 2,82 | 0,01 |
| 40 | MOSPD1 | motile sperm domain containing 1 | 2,2 | 0,01 |
| 41 | C9orf86 |  | 2,1 | 0,011 |
| 42 | EBI3 | Epstein-Barr virus induced 3 | 3,68 | 0,011 |
| 43 | HBEGF | heparin-binding EGF-like growth factor | 2,48 | 0,011 |
| 44 | XLKD1 | lymphatic vessel endothelial hyaluronan receptor 1 | 2,41 | 0,012 |
| 45 | FBXW7 | F-box and WD repeat domain containing 7, | 2,26 | 0,013 |
| 46 | HIST1H2AC | histone cluster 1, H2ac | 2,41 | 0,014 |
| 47 | IGFBP6 | insulin-like growth factor binding protein 6 | 2,04 | 0,015 |
| 48 | C12orf44 |  | 2,12 | 0,016 |
| 49 | IL1R1 | interleukin 1 receptor, type I | 2,12 | 0,017 |
| 50 | MATN3 | matrilin 3 | 2,06 | 0,017 |
| 51 | KLRC3 | killer cell lectin-like receptor subfamily C, member 3 | 3,22 | 0,018 |
| 52 | SERTAD1 | SERTA domain containing 1 | 2,08 | 0,019 |
| 53 | CTHRC1 | collagen triple helix repeat containing 1 | 3,04 | 0,02 |
| 54 | STK17B | serine/threonine kinase 17b | 2,56 | 0,023 |
| 55 | LMCD1 | LIM and cysteine-rich domains 1 | 2,93 | 0,023 |
| 56 | HSPB8 | heat shock 22kDa protein 8 | 2,04 | 0,024 |
| 57 | EMP3 | epithelial membrane protein 3 | 2,24 | 0,024 |
| 58 | CDKN2B | cyclin-dependent kinase inhibitor 2B | 2,46 | 0,024 |
| 59 | PPCS | phosphopantothenoylcysteine synthetas | 2,07 | 0,025 |
| 60 | ASB4 | ankyrin repeat and SOCS box containing 4 | 2,06 | 0,025 |
| 61 | OBFC2A | oligonucleotide/oligosaccharide-binding fold containing 2A | 2,2 | 0,027 |
| 62 | GCNT3 | glucosaminyl (N-acetyl) transferase 3, mucin type | 2,21 | 0,029 |
| 63 | ACTG2 | actin, gamma 2, smooth muscle, enteric | 2,43 | 0,03 |
| 64 | SCFD1 | sec1 family domain containing | 2,22 | 0,032 |
| 65 | ANXA3 | annexin A3 | 2,05 | 0,032 |
| 66 | DUSP1 | dual specificity phosphatase1 | 2,1 | 0,037 |
| 67 | QPCT | glutaminyl-peptide cyclotransferase | 2,2 | 0,037 |
| 68 | CDKN1A | cyclin-dependent kinase inhibitor 1A | 2,02 | 0,041 |
| 69 | GFPT2 | glutamine-fructose-6-phosphate transaminase 2 | 2,14 | 0,043 |
| 70 | LOC124220 | zymogen granule protein 16B | 2,33 | 0,043 |
| 71 | RELB | v-rel avian reticuloendotheliosis viral oncogene homolog B | 2,2 | 0,044 |
| 72 | UPP1 | uridine phosphorylase 1 | 2,13 | 0,045 |
| 73 | LOC731954 |  | 2,01 | 0,046 |
| 74 | CST4 | cystatin S | 2,27 | 0,048 |
| 75 | ATF3 | activating transcription factor 3 | 2,03 | 0,05 |

**Supplementary table-2:** List of ≥2 fold upregulated genes in ATP9A depleted HepG2 cells compared to controls after gene expression microarray (as described for supplementary table-1). List of genes are presented in the order of their significance.
